# The social dynamics of lung cancer talk on Twitter, Facebook and Macmillan.org.uk

**DOI:** 10.1038/s41746-019-0124-y

**Published:** 2019-06-10

**Authors:** Joanna Taylor, Claudia Pagliari

**Affiliations:** eHealth Research Group, Usher Institute for Population Health Sciences and Informatics, University of Edinburgh, Edinburgh, EH8 9AG UK

**Keywords:** Health care, Interdisciplinary studies

## Abstract

People with lung cancer and others affected by the condition are using social media to share information and support, but little is known about how these behaviours vary between different platforms. To investigate this, we extracted posts from Twitter (using relevant hashtags), the Lung Cancer Support Group on Facebook and the Macmillan.org.uk lung cancer discussion forum for a single month. Interaction Process Analysis revealed that all three platforms were used more for giving than seeking information, opinion or suggestions. However, interaction types (including sentiment) varied between platforms, reflecting their digital architectures, user-base and inclusion of a moderator. For example, a higher percentage of information-seeking and sentiment marked the Macmillan.org.uk, compared with Twitter and the Facebook Group. Further analysis of the messages using a four-dimensional typology of social support revealed that emotional and informational support types were most prevalent on the Macmillan.org.uk forum, closely followed by the Facebook Group. Contrary to expectations, Twitter posts showed the most companionship support, reflecting the use of hashtags as user-generated signals of community belonging and interests. Qualitative analysis revealed an unanticipated sub-category of spiritual support, which featured uniquely in the Lung Cancer Support Group on Facebook. There was little evidence of trolling or stigma, although some users remarked that lung cancer was unfairly resourced compared with other cancers. These findings provide new insights about how people affected by lung cancer use social media and begin to elucidate the value of different platforms as channels for patient engagement and support, or as potential research data sources.

## Introduction

Lung cancer is the most common cancer globally, with ~2.09 million cases every year.^1^ There are two main types of primary lung cancer: small cell lung cancer (SCLC), affecting 15% of those diagnosed in the United States, and non-SCLC (NSCLC) affecting 85%.^2^ The severity of this condition varies between Stage 1 and Stage 4, depending on the size of the tumour and whether it has spread. These stages can influence survival rates with fewer than 6% of patients living more than 5 years after diagnosis with Stage 3. Treatment options include but are not limited to surgery, chemotherapy, radiotherapy and laser therapy, and are dependent on the individual. Causes of lung cancer include smoking, passive smoking, exposure to radon and asbestos, air pollution, low immunity and family history, to name but a few.^3^ Although smoking is a known risk factor, 10–15% of people who develop lung cancer are never-smokers and its cause cannot be definitively associated with established environmental risk factors.^4^ Much research has been conducted into the stigma associated with lung cancer^5–7^ and has shown that it is considered more highly stigmatized than other cancers^8,9^ due to self-blame and its causal attribution to smoking.^10,11^ As such, those diagnosed with lung cancer are encouraged to seek support through support groups and online communities, in order to reduce the likelihood of depression.^12^

Social media are online, often mobile, platforms that support the creation and exchange of user-generated content.^13^ They are estimated to have 2.46 billion users worldwide.^14^ Hamm et al.’s^15^ scoping review of studies involving social media use by patients and caregivers reported that discussion forums, online support groups, social networking sites and micro-blogs dominate the research literature, with 11.3% of the identified studies focusing on cancer. Patel et al.’s^16^ systematic review of research on the use of social media in chronic disease, as defined by the Centers for Disease Control, went further by evaluating the clinical outcomes of such technologies. This revealed that Facebook, blogs and Twitter were the most popular social media examined, and that cancer was the most common chronic condition investigated. Although relevant research is fragmented and currently lacking in substantive empirical evidence, existing studies suggest that social media can be used to provide social, emotional or experiential support in chronic disease management and are likely to improve patient care.^16,17^ Psychological support was revealed to be present in the majority of tweets by cancer patients,^18^ whereas a narrative synthesis of cancer patient blogs indicated that users share their diagnosis and treatment journeys online as a means of describing their experiences of health services, informing their health behaviour and in maintaining relationships with others.^19^ Lung cancer is the second most prevalent cancer discussed on Twitter, after breast cancer,^20^ and research has revealed that the majority of relevant tweets focus on treatment and the use of pharmaceutical and research interventions, followed by awareness-raising and prevention/risks.^21^

Health researchers are increasingly using data sourced from social media to understand how members of patient communities interact with each other regarding specific conditions.^22,23^ Single platforms, such as Twitter and Facebook or condition-specific online communities, have dominated previous research. However, a study comparing the use of different social media platforms by patients with Type 1 diabetes revealed variations in the purposes for which these were used, with Twitter mainly used for information and opinion sharing, with little support or empathy, and discussion forums and social networking sites used more often for social interaction and peer support.^24^

We expand on the latter study here, by exploring the types of interaction and support demonstrated on different social media platforms by people affected by lung cancer, with specific reference to lung cancer hashtags on Twitter,^25^ the Lung Cancer Support Group on Facebook^26^ and the lung cancer discussion forum on Macmillan.org.uk.^27^ In doing so, we consider the following research questions: (1) Do people affected by lung cancer use different social media in different ways? and (2) which social media are most successful at encouraging social interaction and support for people affected by lung cancer?

## Results

### Frequency of usage

Table 1 shows the number of lung cancer-related posts extracted from each of the three social media platforms, the total number of people contributing to each platform, the number of replies and the number of relevant English language posts included in the categorization stage. The Twitter hashtags #LCSM and #LungCancer were found to have the highest absolute number of posts (3000 posts over the 1-month period), followed by the Lung Cancer Support Group on Facebook (2644 posts) and the lung cancer discussion forum on Macmillan.org.uk (266 posts).

**Table 1:** Summary of social media posts from October 2017

Source	Number of unique authors	Total sample size	Number of original posts	Number of replies	Number of secondary replies	Number of posts included in IPA categorization stage (%)
Twitter #LCSM and #LungCancer	1056	3000	3000	0	0	2897 (97%)
Facebook Lung Cancer Support Group	844	2644	51	1659	934	2597 (98%)
Macmillan.org.uk lung cancer discussion forum	96	266	51	215	0	266 (100%)

### Contributors

The Twitter hashtags had the highest number of unique authors (1056), compared with the Lung Cancer Support Group on Facebook (844) and the lung cancer discussion forum on Macmillan.org.uk (96). At 2593, the Lung Cancer Support Group on Facebook had the highest number of replies compared with the Macmillan lung cancer discussion forum (215 replies) and the Twitter hashtags (0 replies).

### Types of interaction

**Fig. 1:**
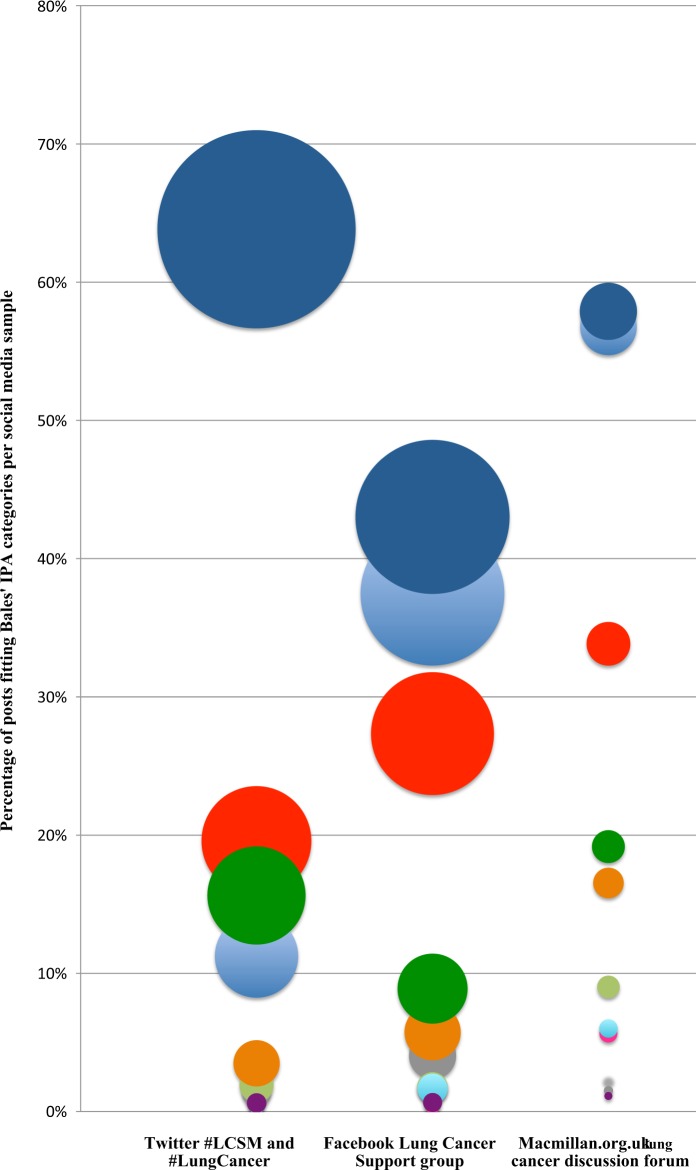
Percentage of posts in the corpus of data from the Twitter lung cancer hashtags, the Lung Cancer Support Group on Facebook and the lung cancer discussion forum on Macmillian.org.uk, and their fit to Bales’ IPA categories. The size of each circle represents the percentage of posts associated with each of Bales’ IPA categories. Each colour represents a different category: light blue for ‘seems friendly’, fuchsia for ‘shows tension release’, grey for ‘agrees’, black for ‘asks for suggestion’, light green for ‘asks for opinion’, orange for ‘asks for information’, dark blue for ‘gives information’, red for ‘gives opinion, dark green for ‘gives suggestion’, lilac for ‘disagrees’, turquoise for ‘shows tension’ and purple for ‘seems unfriendly’

**Fig. 2:**
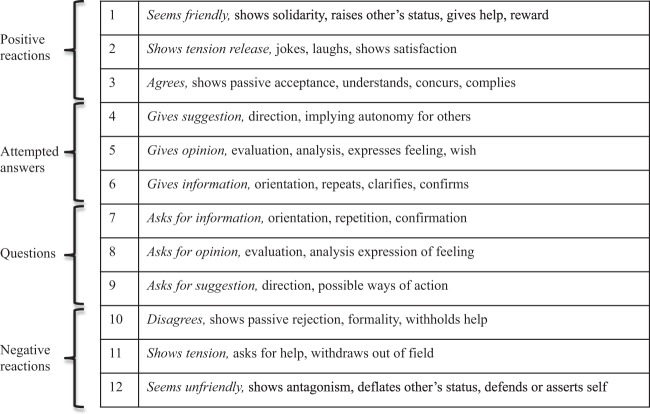
Description of each of Bales’ IPA categories

Table 2 and Fig. 1 show the frequency of posts fitting each of the 12 categories developed by Bales^28^ (Fig. 2) for classifying the type (not content) of interactions taking place in groups, known as Interaction Process Analysis (IPA). Although all three platforms were mainly used to post suggestions, opinions and information, information sharing was the most common use, representing 64% of posts bearing the Twitter lung cancer hashtags, 58% of posts to Macmillan’s lung cancer discussion forum and 43% of posts to the Lung Cancer Support Group on Facebook. Noticeably, fewer posts sought suggestions, opinions and information from other members, although this varied across platforms (5% of Twitter lung cancer hashtag posts, 7% of posts in the Lung Cancer Support Group on Facebook and 28% of the posts on Macmillan’s lung cancer discussion forum). There were also more posts classified as friendly, unfriendly, tension release and showing tension in the sample from Macmillan’s lung cancer discussion forum (56.8% friendly, 6%, shows tension, 5.6% tension release, 1.1% unfriendly) compared with the Lung Cancer Support Group on Facebook (37.5% friendly, 1.7% shows tension, 0.7% unfriendly, 0.4% tension release) and the Twitter lung cancer hashtags (11.3% friendly, 0.6% unfriendly, 0.4% shows tension release, 0.2% tension release), suggesting a greater degree of sentiment expressed in the Macmillan discussion forum. To provide transparency and increase the reproducibility of our analysis, examples of posts from each category and social media platform are provided in Table 3.

**Table 2:** Application of Bales IPA to the lung cancer posts

Source	Total number of posts analysed	Seems friendly	Shows tension release	Agrees	Asks for suggestion	Asks for opinion	Asks for information	Gives information	Gives opinion	Gives suggestion	Disagrees	Shows tension	Seems unfriendly
Twitter #LCSM and #LungCancer	2897	326 (11.3%)	6 (0.2%)	39 (1.3%)	2 (0.1%)	55 (1.9%)	101 (3.5%)	1849 (63.9%)	567 (19.6%)	453 (15.6%)	1 (0.03%)	11 (0.4%)	18 (0.6%)
Facebook Lung Cancer Support Group	2597	973 (37.5%)	10 (0.4%)	105 (4.0%)	1 (0.04%)	45 (1.7%)	149 (5.7%)	1117 (43.0%)	710 (27.3%)	231 (8.9%)	0 (0%)	43 (1.7%)	17 (0.7%)
Macmillan.org.uk lung cancer discussion forum	266	151 (56.8%)	15 (5.6%)	4 (1.4%)	6 (2.3%)	24 (9.0%)	44 (16.5%)	154 (57.9%)	90 (33.8%)	51 (19.2%)	0 (0%)	16 (6.0%)	3 (1.1%)

The categories were not considered mutually exclusive

**Table 3:** Examples of the different categories of post

Source	Seems friendly	Shows tension release	Agrees	Asks for suggestion	Asks for opinion	Asks for information	Gives information	Gives opinion	Gives suggestion	Disagrees	Shows tension	Seems unfriendly
Twitter #LCSM and #LungCancer	‘I’m so saddened & disappointed. My condolences to her loving family & the lung cancer community’ ‘Thanks everyone who walked today’	‘US #1 cancer killer is Lung Cancer: 433 die daily,160 K die yearly! Lungs are sexy, too!’	‘Agreed! Either light up the White House for every cancer or don’t light it up at all! #WhyIsThisPink’	‘How might doctors and other healthcare providers better integrate #yogatherapy into their offerings?’	‘Which lung cancer group(s) do you favour? Why do you like them?’	‘Which Genetic Tests Are Important for Metastatic Lung Cancer Treatment?’	‘#BreakingNews: FDA approves new dosing option for ALUNBRIG (brigatinib) in the US’	‘Let’s not forget that #lungcancer takes more lives than the next four cancers put together. More research clearly needed’	‘For those who’ve lost someone to lung cancer. Join me at the Life & Breath Rally.’	‘This seems wrong’	‘Damn it! xxx died’ ‘This f*kin’ sucks. She so wanted to be in Oahu with her family. I hope she was.’	‘… there is no need to be rude. I am a cancer survivor. I don’t think I brought it on myself & I changed my career to advocate for those who feel their voices are lost…’
Facebook Lung Cancer Support Group	‘Sending love and prayers’ ‘Good luck next week’	‘Nobody is getting out of here alive. Life is a 100% fatal disease.’	‘I agree with you…..hospitals are scary’ ‘I totally agree. Family and friends may be supportive but they don’t understand what we’re going through and feeling like everyone on here’	‘Where and how do all of you find your inner strength? I’m so afraid. Please share what helps you.’	‘Is there hope for Lung cancer? Need some warriors here to post some good news for a change….’	'Does it mean that it’s not advanced NSCLC since my dad tested negative for the PD-L1?’	‘Keytruda did not work for me because I have a ALK mutation in my cancer. I went to different oncologist and she put me on a targeted therapy for that mutation and my tumours are shrinking.’	‘It’s awful but it gets better when the radiation stops. Just work hard on staying hydrated and eating what you can.’	‘Take it day by day and test by test….Stay away from the internet searches and Dr. Google.’		‘we need to STOP the Stigma and not worry about who smokes, smoked or never smoked…it causes so much upset and hard feelings because so many people still ‘assume’ that you had to smoke to get Lung Cancer’	‘SPAM!…You are in violation of the GROUP RULES!’ ‘Comments like that are so ignorant’
Macmillan.org.uk lung cancer discussion forum	‘I’m so so sorry for your loss. Your mom fought and lost but she is now in a better place. I don’t know what to say other than I’m thinking of you and her. Please take care and your time to grieve.’	‘My dad’s taste has completely changed! He’s like a pregnant woman!’	‘My grandad has been having the sweats! Definitely agree. They should make these symptoms more aware. Especially considering how aggressive this cancer is.’	‘My dad has advanced lung cancer. …. Does anyone have any suggestions for good support pillows that helped them or their loved ones?	‘If the 4 chemo sessions I’m going to get are only going to give me a short time extra and in that short time I’m going to feel dreadful, then what’s the point? Does that sound a bit defeatist?’	‘Found out today that if the disease progresses they would consider second line treatments such as Docetaxel or + /− Neratinib. Has anyone any experience of these?’	‘I am brand new and so thankful that I found your site. I went to the ER yesterday for abdominal pain and they did an abdominal CT scan. They found 2 small nodules (2 mm and 3 mm) in one of my lungs. It says 2/3, stable densities suggesting benignity.’	‘Things will get easier when you have a treatment plan and can get your head round it and see the way forward.’	‘You can carry out a search within the Lung group typing some words into the search bar and it should list all discussions containing that text.’ ‘Have you been allocated a Macmillan nurse? If not speak to the oncology department.’		‘The consultant didn’t want to put a time frame on her life expectancy … how the hell do you get through this? How do I explain to my 4 year old when the time comes that grandma isn’t coming over again?’	‘My original post was supposed to provide my Dad with some positive stories and tips…I dont think i can share this thread with him after the last post. Im sympathetic to your situation but just ask you be the same to mine’

### Types of social support

After excluding promotional or news posts (45.1% of the Twitter sample only), data from the three platforms was analysed against the four-dimensional typology of social support.^29–31^ The four social support types are Emotional (offering empathy, concern, affection, love, trust, acceptance, intimacy, encouragement or caring), Instrumental (provision of financial assistance, material goods, services or tangible aid), Informational (provision of advice, guidance, suggestions or useful information to someone), and Companionship (design to give a sense of belonging). The results are summarized in Table 4. These highlight differences between the three social media sources, with informational support being more evident on the discussion forum on Macmillan.org.uk (65.4%) and Lung Cancer Support Group on Facebook (54.7%) compared with the Twitter hashtags (29.1%). Emotional support is also most evident in the lung cancer discussion forum on Macmillan.org.uk (66.9%) compared with Lung Cancer Support Group on Facebook (51.3%) and the Twitter hashtags (5.8%).

**Table 4:** Frequency of the four different functions of social support

Source	Total number of posts analysed	Emotional support	Instrumental support	Informational support	Companionship support	Not considered social support
Twitter #LCSM and #LungCancer	2897	168 (5.8%)	9 (0.3%)	842 (29.1%)	686 (23.7%)	1307 (45.1%)
Facebook Lung Cancer Support Group	2597	1333 (51.3%)	0 (0%)	1421 (54.7%)	71 (2.7%)	0 (0%)
Macmillan.org.uk lung cancer discussion forum	266	178 (66.9%)	0 (0%)	174 (65.4%)	33 (12.4%)	0 (0%)

The categories were not considered mutually exclusive

### Message content and sentiment

**Fig. 3:**
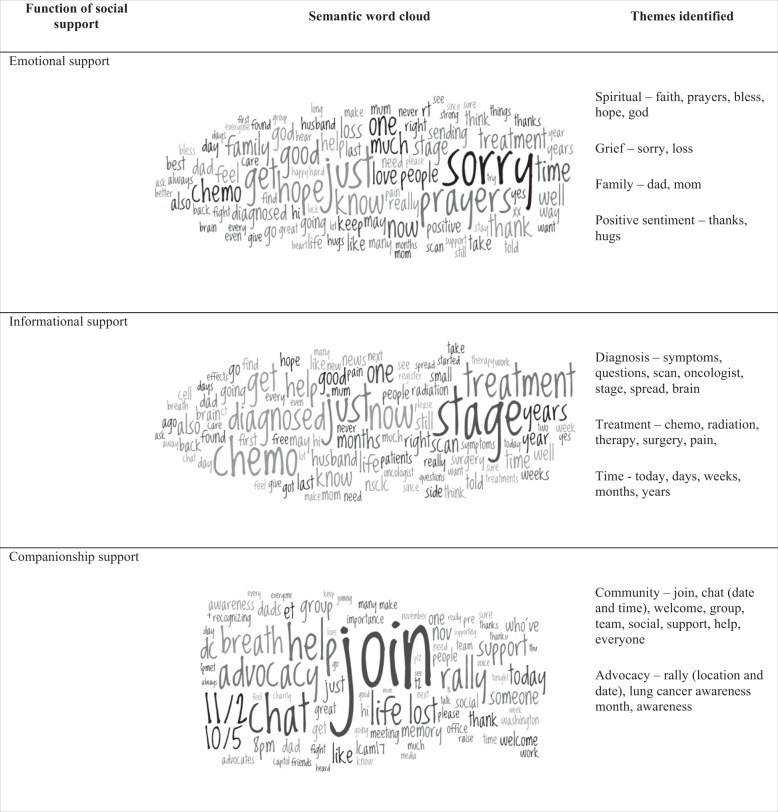
Semantic word clouds visualizing the frequency of words by social support function

In lieu of qualitative analysis, the frequency and co-occurrence of keywords associated with posts in each social support category, from the three social media platforms, were mapped into the semantic ‘word clouds’ shown in Fig. 3. No word cloud was produced for ‘Instrumental’ support, due to the small number of posts in this category. The themes characterizing posts falling into each of the remaining three social support categories are shown in the right-hand column. Emotional support is represented by qualitative themes such as spirituality, grief, family and positive sentiment, whereas informational support is identified by terms related of the diagnosis and treatment of the condition. Keywords relevant to community and advocacy are evident in the category of companionship support.

## Discussion

This descriptive analysis, encompassing a total of 5910 relevant messages posted on Twitter using the hashtags #LCSM and #LungCancer, the Lung Cancer Support Group on Facebook and the Macmillian.org.uk lung cancer discussion forum, identified variations in the use of different social media by people affected by lung cancer. These variations include the nature of interactions within these online communities and the type of social support represented. Across the three platforms, the absolute number of postings in the 1-month observation period was greatest for the Twitter hashtags, followed by the Lung Cancer Support Group on Facebook and then the lung cancer discussion forum on Macmillan.org.uk. However, these raw numbers say little about their value for users.

As noted earlier, the Bales’ IPA highlighted similarities and differences in the type of interactions found on each of the three social media platforms. All are predominantly used for information sharing, with the lung cancer discussion forum on Macmillan.org.uk also showing the most posts expressing sentiment (e.g., friendliness, tension).

There are several possible explanations for these differences. One relates to the digital architectures of the platforms—defined as ‘the technical protocols that enable, constrain and shape user behaviour in a virtual space’.^32^ Twitter is designed for individual micro-blogging (broadcasting), whereas Facebook groups and discussion forums are designed for conversation and sharing. Although Twitter posts may trigger chains of responses, this is coincidental, whereas Facebook groups are explicitly designed for this and include invited members, often mirroring offline social networks.^33^ In our study, high levels of two-way communication were seen in the Lung Cancer Support Group on Facebook (99.8%) and lung cancer discussion forum on Macmillan.org.uk (81%), whereas the sample of Twitter posts contained only original tweets and retweets.

An observation that warrants further investigation, relates to the types of account holders posting to the different social media platforms. In previous analyses of health-related Twitter narratives, 25% of verified accounts belonged to journalists,^33^ 40% to companies and brand accounts, and 15–20% were bots.^34^ In our study, organizations (e.g., pharmaceutical companies, charities), patient advocacy groups, research institutions and news outlets appear to use Twitter to disseminate health information to the general public. In contrast, the Lung Cancer Support Group on Facebook and the discussion forum on Macmillan.org.uk were chiefly designed for people affected by lung cancer and due to the presence of community moderators, function as online support groups and enable a greater degree of interaction.

Community moderators or administrators play an influential role in online communities,^35,36^ as was the case in the closed (members only) Lung Cancer Support Group on Facebook and the lung cancer discussion forum on Macmillan.org.uk where their presence created greater understanding of the community rules and expectations. In the case of the Lung Cancer Support Group on Facebook, group members were asked to agree to a code of conduct, requiring them to respect others, avoid foul language, focus on lung cancer, not seek medical advice, refrain from advertising or fund-raising and never block an admin, or face removal. As well as providing a ‘safe’ place for people to share information and experiences, moderators of the Macmillan.org.uk lung cancer discussion forum responded to posts that had not yet received a comment from other community members. Examples of this include ‘sorry to hear your news, and I’m also sorry you’ve not had a reply yet’. In responding to this post, the moderator was able to increase the visibility of the post in an attempt to garner a response. In contrast, moderation of content posted on Twitter is absent, except during the 1 hour pre-scheduled #LCSM group discussion that takes place every 2 weeks.^37^ The moderator in these cases is there to greet participants and facilitate a discussion based on predefined questions (e.g., ‘I will announce four topic questions (T1 T2 etc). Pls label your answers with T1, T2, etc to make transcript easier to follow #lcsm’).

Findings also revealed that posts by any user that are similar to an online survey would generate the most responses on Facebook, as was also the case in a previous diabetes study.^24^ Examples of this include: ‘how old was everyone when they were first diagnosed?’ (609 responses), ‘this may be a really dumb question: can someone survive lung cancer?’ (228 responses) and ‘anyone got a husband or wife that won’t give up smoking?’ (73 responses).

In summary, although the Lung Cancer Support Group on Facebook and lung cancer discussion forum on Macmillan.org.uk were moderated, this is limited in Twitter to pre-scheduled group discussions. We find that Twitter was the most actively used social media in terms of volume of posts, whereas Facebook achieved the highest percentage response rate and interaction. The differences in digital architecture, in turn contribute to the variations in social interaction and support for people affected by lung cancer.

We observed several interesting differences in the social interaction represented in the posts on these three social media platforms. As previously noted, the lung cancer hashtags on Twitter are mainly used to disseminate information (64%) and opinion (20%) in line with research describing Twitter as a mass communication and broadcasting tool^38^ with the majority of active narratives involving two or fewer users.^39^ Findings from this study are similar in nature to that of the Type 1 diabetes comparative study from which we drew inspiration.^24^

The positive correlation between social support and health is widely accepted among the public health and psychology research communities.^40^ In addition to types of functional interaction shown in the posts, our analysis revealed differences in the types of social support that are being sought and offered by users of different social media. All content posted in the Macmillan.org.uk lung cancer discussion forum and in the Lung Cancer Support Group on Facebook was associated with at least one of the functions of social support whilst 43.6% of tweets were not and in most cases can be considered either news reports (e.g., ‘FDA Approves Higher-Dose Tablet of Brigatinib for NSCLC’), promotional messages (e.g., ‘Get your #lungcancer swag for #LCAM17 this Nov. T-shirts, tank tops, sunglasses, bracelets and more!’) or advocacy-related (e.g., ‘#433aday Lung cancer kills 433 Americans a day. We need better funding for research now’) in content.

Informational support, as already noted, is the provision of information aimed at supporting a member or members of the social network, often in response to a statement of distress or a request for help. Although this overlaps with Bales’ IPA categories of ‘information giving’ they are not synonymous. The lung cancer discussion forum on Macmillan.org.uk produced the highest percentage of informational support posts (65.7%), followed by the Lung Cancer Support Group on Facebook (54%), the #LungCancer on Twitter (40.7%) and the #LCSM on Twitter (15.4%). Posts such as ‘Looking for advice. Mum is in later stages of stage 4 lung cancer….In the last 4 days mum has stopped eating and can only get a small amount of fluids in her…Not sure what to do or what to expect now’ and ‘can radio be used after Keytruda? Can it be keytruda + radio?’ illustrate the type of informational support sought. Posts classified as informational support are represented in words related to the diagnosis, treatment and progression of the condition over time, a finding which aligns somewhat to that from Tsuya et al.^19^ study into whether cancer patients tweet.

The lung cancer discussion forum on Macmillan.org.uk produced the highest percentage of emotional support posts (67.2%), followed by the Lung Cancer Support Group on Facebook (50.6%), the lung cancer hashtags on Twitter (5.8%), contradicting findings from previous research that revealed that the majority of tweets posted by cancer patients focused on psychological support.^18^ Emotional support is represented in posts such as ‘I am so sorry to hear this…you are in my thoughts’ and ‘It really is so hard…I’m full of hurt and anger. Just seems so unfair’. Words associated with emotional support include spiritual and religious terms (e.g., faith, god, hope, prayers), grief (e.g., sorry, loss), family and positive sentiment (e.g., thanks, hugs).

Given the severity of lung cancer, its treatment and the life-threatening nature of the condition, it is not surprising that spiritual and existential beliefs are represented in the social media data^41^; Lung Cancer Support Group on Facebook (14.5%), lung cancer discussion forum on Macmillan.org.uk (3.4%) and lung cancer hashtags on Twitter (0.7%). Identified as one of the modifiable dimensions of the patient experience, much research has been conducted into the role spirituality and faith plays in the illness trajectories of lung cancer patients.^42–44^ Its manifestation in social media, however, is a relatively new field^45–47^ with little empirical evidence of how social media platforms differ in this context. Findings from our study suggest that spirituality is significantly more prevalent on Facebook rather than Twitter and further research is warranted to test this hypothesis.

Surprisingly, the Twitter lung cancer hashtags indicated the highest percentage of companionship support (23.7%) compared with the lung cancer discussion forum on Macmillan.org.uk (12.5%) and Lung Cancer Support Group on Facebook (2.7%). Words associated with this form of social support include those related to joining online conversations and physical events, manifesting themself in posts such as ‘Sending so much love to all of you. Love my tribe. Let’s do this’, ‘Anyone can do something…A handful of us started #LCSM’ and ‘Join us on November 2 in Washington DC’. The higher percentage of companionship support posts on Twitter, may be attributed to the use of the #LCSM as a means of forming topic communities where large groups of users, who do not need to be connected through existing ‘follower’ networks, can interact within the constraints of Twitter’s digital architecture.^48^ The #LCSM hashtag was created with the intention to ‘unite patients, caregivers, advocates, healthcare providers and researchers to discuss ways to improve lung cancer diagnosis, treatment, research, patient outcomes, caregiving, information sharing and public support’. It connects those participating in the pre-scheduled online discussion and is supported by posts such as ‘If anyone is just lurking tonight, please blank tweet the hashtag #LCSM so we know you’re out there. We’re a friendly bunch’ and ‘Remember to include #lcsm in your tweets…’. This suggests that through the use of hashtags and followers^49^ Twitter can provide a source of social and community support to people affected by lung cancer in knowing that they are not alone and in building self-esteem, confidence and social validation.^50^

Instrumental support was not present in any of the posts in the Lung Cancer Support Group on Facebook nor the lung cancer discussion forum on Macmillan.org.uk. It was however present on Twitter (0.3%) as users, often from the United States, requested financial support to fund their treatment ‘Help me complete #LungCancer treatment #Donate #crowdfund Please retweet!’

Other topics that are notably scarce in our samples include stigmatization and trolling.

Although health-related stigmatization on social media has been reported in other research^51^ and in specific about lung cancer,^52^ here it was seen in only 1.8% of Twitter hashtags, 0.5% of posts to the Lung Cancer Support Group on Facebook and not at all in the lung cancer discussion forum on Macmillan.org.uk (0%). Examples showing how it was manifested include: ‘A lot of people with cancer are afraid to talk about it, especially Lung Cancer, since some will just assume you smoke and you did it to yourself’ (Facebook user), ‘Lung cancer is the biggest killer yet there is no education on it. I do believe it is the stigma of smoking that is associated with it…it is the first thing people say to me’ (Facebook user) and ‘We’re fighting stigma that holds lung cancer back from broad public sympathy despite being biggest cancer killer’ (Twitter user).

Trolling is defined as ‘the practice of behaving in a deceptive, destructive, or disruptive manner in a social setting on the internet with no apparent instrumental purpose’.^53^ Unfriendly social interactions were represented by <1% of posts in the corpus of data from the three social media platforms; contrasting with a recent analysis showing that 24% of online trolling incidents are associated with health related topics.^54^

Limitations in our study reflect the self-selective nature of contributors, as a social media account is required in order to author a post. Given the ‘digital health divide’^55^ and recent statistics suggesting that most people diagnosed with lung cancer are 65 years or older,^56^ the sample does not perfectly represent the population of people diagnosed and living with this condition. Analyses of social media data has its own limitations due to the presence of misinformation and a lack of provenance of account holders,^23^ a topic that has recently attracted media attention due to use of bot factories to boost followers and the creation of ‘fake news’.^57^ In addition, the cross sectional nature of the study did not take into account variations in activity that may have occurred over time, which can be useful for examining evolving narratives during cancer progression.^49^ Althoughthe data from the Lung Cancer Support Group on Twitter and discussion forum on Macmillan.org.uk were extracted manually, the Symplur Transcript and Analytics tool was used to extract the Twitter data, making the sampling less easy to verify. Other automated social media mining techniques and natural language processing tools are available; however, in some cases these require software and data access licenses, and can vary in their accuracy and effectiveness due to the scope and quality of data available and the types of social media for which they are suited.

In conclusion, our findings, based on a systematic analysis of comparable lung cancer posts on three social media platforms, indicate that although all three are being used to disseminate information about lung cancer, the Lung Cancer Support Group on Facebook and lung cancer discussion forum on Macmillan.org.uk, by virtue of their digital architecture, user-base and self-moderating communities, are more successful in their utility for social interaction and emotional and informational social support. While the sample derived from Twitter hashtags contained fewer posts related to social support across the four categories, posts tagged #LCSM showed the greatest degree of companionship support, revealing how the affordances of this platform can be shaped by its users through the use of a community hashtag. Further analysis also revealed an unanticipated sub-category of spiritual support, which featured uniquely in the Lung Cancer Support Group on Facebook and warrant additional research, as well as limited evidence of resentment about the comparative stigmatization of lung cancer compared with other types of cancer.^10^

These findings provide tentative insights into the social and supportive value of different social media, and show how interactions may be shaped both by the configuration and moderation of the platforms and by users self-organizing around groups or hashtags. They suggest that healthcare providers and policy makers wishing to provide supportive interventions via social media, including the use of social media to reduce stigma,^58^ should prioritize community-based forums over general social media broadcasting. Likewise, academics and public health analysts wishing to study lung cancer via social media should carefully consider the types of data likely to appear on different platforms and its suitability for answering their research questions (e.g., whether through depth or volume). Importantly, the results also provide empirical evidence that people affected by lung cancer, and those supporting them (e.g., healthcare teams, family, carers, clergy), should consider online communities as an additional source of social support during times of crisis.

Although hand searching and content analysis has proven effective in identifying and comparing the key types and expressions of social support for lung cancer manifested on these social media platforms further research is needed to unpick, replicate and extend these findings with larger samples of data. Automated data mining and natural language processing techniques enable the capture and analysis of much larger volumes of data across multiple social media platforms, offering potential to create a greater degree of precision^59^ when combined with appropriate qualitative analysis. Nevertheless, it is essential that third party users, including healthcare providers, acknowledge the sensitivity of users’ data, albeit it has been voluntarily placed in the public domain, since current research ethics guidelines governing use of aggregated social media data remain inconsistent.^60^

## Methods

### Study design

For the purposes of this study we chose three social media platforms, which have different characteristics. Twitter and Facebook are general platforms for information sharing and social networking. In the case of Twitter, communities and topics are often collectively organized around a set of hashtags, which can then be searched for to understand particular issues, as in this study. Users of Facebook can set up discussion groups focused on certain topics, including lung cancer. Specialist organizations, such as Macmillan Cancer Support, may also set up condition-specific online discussion forums, where users can contribute content and others can comment. Users of social media must create an online profile and account ID when registering for these platforms. In doing so, it is within their gift to disclose personal information if they so choose. This variability results in some users being more easily identifiable as a patient, carer or family member than others. Given this incompleteness of profile data, our analysis treats patients and their carers or family members as one group of people affected by lung cancer.

To compare lung cancer-related interactions on different social media we took a multi-stage approach: (a) extracting and screening posts appearing in each social media platform, (b) classifying posts using Bales’ IPA,^28^ (c) categorizing posts according to the four functions of social support and (d) analyzing the 100 most frequent keywords to generate semantic ‘word clouds’ (using Wordle^61^) to visualize the frequency of terms used in posts associated with each form of social support.

### Data sources and screening

Twitter: a sample of 3000 tweets was extracted using Symplur’s Transcript and Analytics tool in December 2017, using the #LungCancer and #LCSM hashtags.^62^ These hashtags were selected due to their specific relevance to the condition and as a means of reducing the amount of ‘noise’ presented in the data. The sample of Twitter data extracted and analysed in this study does not encompass the universe of all Twitter data available. Due to the limitations of the Transcript and Analytics tool, the sample size is limited to 1500 tweets per hashtag and the time frame for the extracted tweets was between 11:55 p.m. on 30 September 2017 and 01:00 a.m. on 1 November 2017. Although the transparency of Symplur’s search and sampling algorithms has been criticized, its use in over 280 published research articles^63^ provided justification for its use in this study. The data extracted included the Twitter account ID and the text in the tweet. Retweets and tweets that were not in English were excluded from the categorization stage. Url links and images included in the tweets were not captured or reviewed during the screening.

Facebook: using the Facebook search functionality, we searched for lung cancer and in doing so identified the largest lung cancer community available on Facebook. Known as the Lung Cancer Support Group, this community was established in 2015 and is a closed group for lung cancer patients, survivors, caregivers and loved ones. Closed groups are members only groups, where the group’s existence is visible to anyone with a Facebook account. Membership of the group, however, is granted through the group administrators. Access to the Lung Cancer Support Group was granted via correspondence sent to the group administrators explaining the intent behind our request. As of 30 December 2017, it had 7975 members and on this same date all wall posts and replies that were posted between 1 and 31 October 2017 were identified by using the search functionality and stipulating the posts could be posted by ‘anyone’, ‘anywhere’ and in ‘October 2017’. A sample of the original post and any associated replies, along with the author and the date of post were manually extracted for further analysis. Posts that were not in English were excluded from the categorization stage. Url links and images included in the posts were not captured or reviewed during the screening.

Macmillan.org.uk**:** Macmillan Cancer Support is a UK based charity that was founded in 1911 and provides specialist healthcare, information, financial and emotional support to people affected by cancer. It has an online community of over 100,000 members^64^ spanning all forms of the condition. The lung cancer discussion forum on Macmillan.org.uk was identified through the search functionality of the online community homepage and the list of discussion threads was then filtered based on the start date 1 October 2017. All original posts and replies posted between 1 and 31 October 2017 were identified and manually extracted. The total sample of 266 posts was included in the categorization stage.

### Categorization of posts by the application of Bales’ IPA and the social support taxonomy

The same sample of lung cancer posts included in the categorization stage were reviewed against the 12 categories of group interaction defined in Bales’ IPA (Fig. 2) as well as the four functions of social support (i.e., emotional, informational, instrumental, companionship). Each post was considered a single unit of interaction and the categories were not considered mutually exclusive when applied to the sample of posts.

### Analysis of keywords

The content of lung cancer posts meeting each category of social support was analysed by inputting the data into the text visualization tool Wordle.^61^ The resulting semantic word clouds map the frequency and co-occurrence of different terms appearing in a corpus of text and can be used by researchers to compare the topics and sentiment appearing in different text.^65^ The word clouds produced in this study represent the top 100 words with the highest frequency of occurrence, in alphabetical order. Hashtags and terms such as ‘lung’, ‘cancer’ and ‘lungcancer’ were removed, as these had already been used to screen the social media posts for inclusion in the study.

### Ethical considerations and informed consent

Although the data available on Twitter, public Facebook pages and the Macmillan.org.uk discussion forum exist in the public domain and can therefore be mined for research purposes without the need to obtain explicit informed consent from the data subjects,^66^ ethical research conduct and digital etiquette are nevertheless required. As recommended by a recent review on the readiness of ethics guidelines to address research involving the secondary use of social media data,^60^ we applied relevant sections from guidance developed by the UK Economic and Social Research Council,^67^ the British Psychological Society^68^ and the Association of Internet Researchers.^69^ In the case of the closed Facebook group, access and agreement to extract and analyse the posts was requested from the group administrators. In order to protect the anonymity of the post authors, their account ID and any reference to other account IDs (e.g., the use of @) were removed). Ethics approval was provided by the University of Edinburgh’s Institutional Review Board.

### Reporting summary

Further information on research design is available in the Nature Research Reporting Summary.

## Supplementary information

Reporting Summary

## Data Availability

The data used in this study is publicly available on Twitter, Facebook and Macmillan.org.uk.

## Supplementary information

**Supplementary Information** accompanies the paper on the *npj Digital Medicine* website (10.1038/s41746-019-0124-y).
