# Supplementary material for: The social dynamics of lung cancer talk on Twitter, Facebook and Macmillan.org.uk
Source: NPJ Digit Med. 2019 Jun 10;2:51. doi: 10.1038/s41746-019-0124-y (PMC6557847; doi:10.1038/s41746-019-0124-y)
Supplement: Supplementary file 1 — Reporting Summary [file 41746_2019_124_MOESM1_ESM.pdf]

nature research

Corresponding author(s): DBPR-NPJDIGITALMED-00215

Last updated by author(s): 2019-03-31

Reporting Summary

Nature Research wishes to improve the reproducibility of the work that we publish. This form provides structure for consistency and transparency in reporting. For further information on Nature Research policies, see Authors & Referees and the Editorial Policy Checklist.

Please do not complete any field with "not applicable" or n/a. Refer to the help text for what text to use if an item is not relevant to your study.
For final submission: please carefully check your responses for accuracy; you will not be able to make changes later.

Statistics

For all statistical analyses, confirm that the following items are present in the figure legend, table legend, main text, or Methods section.

n/a	Confirmed	
☐	☑	The exact sample size (n) for each experimental group/condition, given as a discrete number and unit of measurement
☐	☑	A statement on whether measurements were taken from distinct samples or whether the same sample was measured repeatedly
☑	☐	The statistical test(s) used AND whether they are one- or two-sided<br>*Only common tests should be described solely by name; describe more complex techniques in the Methods section.*
☑	☐	A description of all covariates tested
☑	☐	A description of any assumptions or corrections, such as tests of normality and adjustment for multiple comparisons
☑	☐	A full description of the statistical parameters including central tendency (e.g. means) or other basic estimates (e.g. regression coefficient) AND variation (e.g. standard deviation) or associated estimates of uncertainty (e.g. confidence intervals)
☑	☐	For null hypothesis testing, the test statistic (e.g. F, t, r) with confidence intervals, effect sizes, degrees of freedom and P value noted<br>*Give P values as exact values whenever suitable.*
☑	☐	For Bayesian analysis, information on the choice of priors and Markov chain Monte Carlo settings
☑	☐	For hierarchical and complex designs, identification of the appropriate level for tests and full reporting of outcomes
☑	☐	Estimates of effect sizes (e.g. Cohen's d, Pearson's r), indicating how they were calculated

Our web collection on statistics for biologists contains articles on many of the points above.

Software and code

Policy information about availability of computer code

Data collection	*Symplur Transcript and Analytics tool for extraction of tweets.*
Data analysis	*Microsoft Excel.*

For manuscripts utilizing custom algorithms or software that are central to the research but not yet described in published literature, software must be made available to editors/reviewers. We strongly encourage code deposition in a community repository (e.g. GitHub). See the Nature Research guidelines for submitting code & software for further information.

Data

Policy information about availability of data

All manuscripts must include a data availability statement. This statement should provide the following information, where applicable:

- Accession codes, unique identifiers, or web links for publicly available datasets
- A list of figures that have associated raw data
- A description of any restrictions on data availability

The data used in this study is publicly available on Twitter, Facebook and Macmillan.org.uk.

Field-specific reporting

Please select the one below that is the best fit for your research. If you are not sure, read the appropriate sections before making your selection.

☐ Life sciences ☑ Behavioural & social sciences ☐ Ecological, evolutionary & environmental sciences

Life sciences study design

All studies must disclose on these points even when the disclosure is negative.

Sample size	*Not applicable*
Data exclusions	*Not applicable*
Replication	*Not applicable*
Randomization	*Not applicable*
Blinding	*Not applicable*

Behavioural & social sciences study design

All studies must disclose on these points even when the disclosure is negative.

Study description	*Collection, classification and frequency analysis of qualitative data posted about lung cancer on three different social media platforms.*
Research sample	*Posts made by registered users of three social media platforms i.e. Twitter, Lung Cancer Support Group on Facebook and lung cancer discussion forum on Macmillan.org.uk.*
Sampling strategy	*Sampling of posts on the social media platforms: For the sample of posts from Twitter, the sampling was done through the use of hashtags and use of Symplur's Transcript and Analytics tool, for which the sampling procedure is unknown. For the Lung Cancer Support Group on Facebook and lung cancer discussion forum on Macmillan.org.uk, all posts were included in the sample from the one month period.*
Data collection	*Data was collected manually by one researcher using a computer. The researcher was not blind to the experimental condition that was being investigated.*
Timing	*Data posted during October 2017, was collected from all three social media platforms in December 2017.*
Data exclusions	*No data was excluded during the collection stage. Non English language posts were excluded during the categorisation stage.*
Non-participation	*No participants dropped out or declined to participate.*
Randomization	*Participants were not allocated to experimental groups other than social media platform from which the data was sourced.*

Ecological, evolutionary & environmental sciences study design

All studies must disclose on these points even when the disclosure is negative.

Study description	*Not applicable*
Research sample	*Not applicable*
Sampling strategy	*Not applicable*

Data collection	*Not applicable*
Timing and spatial scale	*Not applicable*
Data exclusions	*Not applicable*
Reproducibility	*Not applicable*
Randomization	*Not applicable*
Blinding	*Not applicable*

Did the study involve field work? ☐ Yes ☑ No

Field work, collection and transport

Field conditions	*Not applicable*
Location	*Not applicable*
Access and import/export	*Not applicable*
Disturbance	*Not applicable*

Reporting for specific materials, systems and methods

We require information from authors about some types of materials, experimental systems and methods used in many studies. Here, indicate whether each material, system or method listed is relevant to your study. If you are not sure if a list item applies to your research, read the appropriate section before selecting a response.

Materials & experimental systems

n/a	Involved in the study	
☑	☐	Antibodies
☑	☐	Eukaryotic cell lines
☑	☐	Palaeontology
☑	☐	Animals and other organisms
☐	☑	Human research participants
☑	☐	Clinical data

Methods

n/a	Involved in the study	
☑	☐	ChIP-seq
☑	☐	Flow cytometry
☑	☐	MRI-based neuroimaging

Antibodies

Antibodies used	*Not applicable*
Validation	*Not applicable*

Eukaryotic cell lines

Policy information about cell lines

Cell line source(s)	*Not applicable*
Authentication	*Not applicable.*
Mycoplasma contamination	*Not applicable*

Commonly misidentified lines (See ICLAC register)	*Not applicable*

Palaeontology

Specimen provenance	*Not applicable*
Specimen deposition	*Not applicable*
Dating methods	*Not applicable*

☐ Tick this box to confirm that the raw and calibrated dates are available in the paper or in Supplementary Information.

Animals and other organisms

Policy information about studies involving animals; ARRIVE guidelines recommended for reporting animal research

Laboratory animals	*Study did not involve laboratory animals.*
Wild animals	*Not applicable*
Field-collected samples	*Not applicable*
Ethics oversight	*Not applicable*

Note that full information on the approval of the study protocol must also be provided in the manuscript.

Human research participants

Policy information about studies involving human research participants

Population characteristics	*See above*
Recruitment	*Participants have a user account for the social media platforms included in the study.*
Ethics oversight	*Relevant ethical guidelines were applied and approval was provided by the University of Edinburgh IRB.*

Note that full information on the approval of the study protocol must also be provided in the manuscript.

Clinical data

Policy information about clinical studies

All manuscripts should comply with the ICMJE guidelines for publication of clinical research and a completed CONSORT checklist must be included with all submissions.

Clinical trial registration	*Not applicable*
Study protocol	*Not applicable*
Data collection	*Not applicable*
Outcomes	*Not applicable*

ChIP-seq

Data deposition

☐ Confirm that both raw and final processed data have been deposited in a public database such as GEO.

☐ Confirm that you have deposited or provided access to graph files (e.g. BED files) for the called peaks.

Data access links<br>*May remain private before publication.*	*Not applicable*
Files in database submission	*Not applicable*
Genome browser session<br>(e.g. UCSC)	*Not applicable*

Methodology

Replicates	*Not applicable*
Sequencing depth	*Not applicable*
Antibodies	*Not applicable*
Peak calling parameters	*Not applicable*
Data quality	*Not applicable*
Software	*Not applicable*

Flow Cytometry

Plots

Confirm that:

☐ The axis labels state the marker and fluorochrome used (e.g. CD4-FITC).

☐ The axis scales are clearly visible. Include numbers along axes only for bottom left plot of group (a 'group' is an analysis of identical markers).

☐ All plots are contour plots with outliers or pseudocolor plots.

☐ A numerical value for number of cells or percentage (with statistics) is provided.

Methodology

Sample preparation	*Not applicable*
Instrument	*Not applicable*
Software	*Not applicable*
Cell population abundance	*Not applicable*
Gating strategy	*Not applicable*

☐ Tick this box to confirm that a figure exemplifying the gating strategy is provided in the Supplementary Information.

Magnetic resonance imaging

Experimental design

Design type	*Not applicable*
Design specifications	*Not applicable*
Behavioral performance measures	*Not applicable*

Acquisition

Imaging type(s): *Not applicable*

Field strength: *Not applicable*

Sequence & imaging parameters: *Not applicable*

Area of acquisition: *Not applicable*

Diffusion MRI ☐ Used ☑ Not used

Parameters: *Not applicable.*

Preprocessing

Preprocessing software: *Not applicable*

Normalization: *Not applicable*

Normalization template: *Not applicable*

Noise and artifact removal: *Not applicable*

Volume censoring: *Not applicable*

Statistical modeling & inference

Model type and settings: *Not applicable*

Effect(s) tested: *Not applicable*

Specify type of analysis: ☐ Whole brain ☐ ROI-based ☐ Both

Anatomical location(s): *Not applicable*

Statistic type for inference (See Eklund et al. 2016): *Not applicable.*

Correction: *Not applicable*

Models & analysis

n/a	Involved in the study
☑	☐ Functional and/or effective connectivity
☑	☐ Graph analysis
☑	☐ Multivariate modeling or predictive analysis

Functional and/or effective connectivity: *Not applicable*

Graph analysis: *Not applicable*

Multivariate modeling and predictive analysis: *Not applicable*

This checklist template is licensed under a Creative Commons Attribution 4.0 International License, which permits use, sharing, adaptation, distribution and reproduction in any medium or format, as long as you give appropriate credit to the original author(s) and the source, provide a link to the Creative Commons license, and indicate if changes were made. The images or other third party material in this article are included in the article's Creative Commons license, unless indicated otherwise in a credit line to the material. If material is not included in the article's Creative Commons license and your intended use is not permitted by statutory regulation or exceeds the permitted use, you will need to obtain permission directly from the copyright holder. To view a copy of this license, visit http://creativecommons.org/licenses/by/4.0/
